# Participant Experiences in the Environmental Determinants of Diabetes in the Young Study: Common Reasons for Withdrawing

**DOI:** 10.1155/2016/2720650

**Published:** 2015-11-22

**Authors:** Barbro Lernmark, Kristian Lynch, Judith Baxter, Roswith Roth, Tuula Simell, Laura Smith, Ulrica Swartling, Suzanne Bennett Johnson, TEDDY Study Group

**Affiliations:** ^1^Department of Clinical Sciences, Lund University, CRC, Jan Waldenströms Gata 35, Skåne University Hospital (SUS), 20502 Malmö, Sweden; ^2^Health Informatics Institute, University of South Florida, 3650 Spectrum Boulevard, Suite 100, Tampa, FL 33612, USA; ^3^Barbara Davis Center for Childhood Diabetes, School of Medicine, University of Colorado Denver-AMC, 1775 Aurora Court, Aurora, CO 80045, USA; ^4^Colorado School of Public Health, Department of Community and Behavioral Health, University of Colorado Denver-AMC, 13001 E. 17th Place, Aurora, CO 80045, USA; ^5^Institute of Diabetes Research, Helmholtz Center Munich and Clinic on the right of Isar, Technical University Munich, Research Group Diabetes e.V., Ingolstaedter Landstrasse 1, 85764 Neuherberg, Germany; ^6^Department of Pediatrics, University of Turku, Kiinamyllynkatu 4-8, 20100 Turku, Finland; ^7^Department of Medical Humanities and Social Sciences, Florida State University College of Medicine, 1115 West Call Street, Tallahassee, FL 32306, USA; ^8^Pacific Northwest Diabetes Research Institute, 720 Broadway, Seattle, WA 98122, USA; ^9^Center for Biotechnology and Genomic Medicine, Medical College of Georgia, Georgia Regents University, 1120 15th Street, CA-4123, Augusta, GA 30912, USA

## Abstract

*Background*. To characterize participant reasons for withdrawing from a diabetes focused longitudinal clinical observational trial (TEDDY) during the first three study years. *Methods*. 8677 children were recruited into the TEDDY study. At participant withdrawal staff recorded any reason parents provided for withdrawal. Reasons were categorized into (1) family characteristics and (2) protocol reasons. Families who informed staff of their withdrawal were classified as active withdrawals (AW); families without a final contact were considered passive withdrawals (PW). *Results*. Withdrawal was highest during the first study year (*n* = 1220). Most families were AW (*n* = 1549; 73.4%). PW was more common in the United States (*n* = 1001; 37.8%) and among young mothers (*p* = 0.001). The most frequent protocol characteristic was blood draw (55%) and the most common family reason was not having enough time (66%). The blood draw was more common among female participants; being too busy was more common among males. Both reasons were associated with study satisfaction. *Conclusions*. Results suggest that, for families of children genetically at risk for diabetes, procedures that can be painful/frightening should be used with caution. Study procedures must also be considered for the demands placed on participants. Study satisfaction should be regularly assessed as an indicator of risk for withdrawal.

## 1. Introduction

The Environmental Determinants of Diabetes in the Young (TEDDY) study is a multicenter longitudinal clinical observational trial studying the natural history of the development of type 1 diabetes (T1DM) in children. Soon after birth, children were tested for HLA conferred genetic risk for T1DM. Children with the highest genetic risk were invited to participate in TEDDY. The purpose of the TEDDY study is to identify environmental factors that trigger autoimmunity and TIDM [1].

The success of longitudinal research studies, investigating important factors that contribute to T1DM, like TEDDY, is dependent upon study retention. It is important to investigate what study and psychosocial characteristics prompt families to leave a study in order to (1) implement possible preventive actions to increase retention and (2) design future longitudinal studies for this at-risk T1DM population in ways that enhance study retention. Although this topic is critical for the success of longitudinal trials, the extant literature is somewhat sparse. In fact, only 55% percent of pediatric trials report refusal or withdrawal reasons based on a recent literature review [2]. Previous work within more varied pediatric populations and interventional studies (e.g., T1DM, asthma, and obesity intervention trials) has suggested that a number of sociodemographic factors (e.g., older child age, minority status, and lower income) and psychological factors (e.g., greater depression and lower quality of life) were related to study withdrawal [3–6]. However, these findings from intervention studies in chronic illness populations may not be fully applicable to the TEDDY at-risk for T1DM population. Further, previous studies have tended to focus on existing characteristics of participants who do not complete a study rather than directly ascertaining reasons for withdrawal from the participants themselves.

A longitudinal study similar to the TEDDY study reported that logistical matters like blood sampling and lack of time were the reasons most often mentioned by families who withdrew [7]. In the TEDDY population, we have reported that characteristics of the study protocol, like blood draws, and family factors, like being too busy, were the primary reasons families did not join the study [8]. Study enrollment was associated with sociodemographic factors such as whether the child had a mother, father, and/or sibling with T1DM (first degree relative (FDR)), had an older mother, was a singleton, or had a sibling already enrolled in the study. Enrollment rates differed between the TEDDY countries, with a larger proportion of parents recruited from the European countries [8].

In other prior works, we identified predictors of withdrawal during the first year (up to the 15 months' visit) of TEDDY among families from the general population (GP) who had no immediate family member with T1DM [9]. Study withdrawal was more common if the mother was young, the father did not participate, or the study child was female. Also, mothers of children who withdrew were more likely to report smoking during pregnancy, abstaining from alcohol, and reducing their work hours or not working at all during pregnancy. Mothers who withdrew were also more likely to fail to complete items on study questionnaires and to underestimate their child's TIDM risk. Among mothers with accurate perceptions of their child's T1DM risk, high maternal anxiety was associated with study withdrawal [9]. This information was used to identify families at high risk for leaving the TEDDY study in the first year; these families were then provided with an intervention to promote retention [10]. While factors associated with withdrawal could be used to screen for families at risk of dropout, the effectiveness of interventions aimed at improving the retention of participants can also be influenced by the families' specific reasons for withdrawing. Thus, in this study we examined data from all families who left TEDDY (both GP and FDR) during the first three study years, including sociodemographic and psychosocial variables, and their reasons for opting out of the study.

## 2. Methods

### 2.1. TEDDY Study and Data

The TEDDY study has centers in four countries (Finland, Germany, Sweden, and the United States) and is supported by the National Institutes of Health (NIH). The study protocol includes study visits every three months from 3-4 months of age until the child is four years old and biannually thereafter. The study protocol includes blood draws, nasal swabs, height and weight measurements, and parental interviews where aspects of the child's health are recorded together with different types of life events. At regular intervals TEDDY parents fill out questionnaires with demographic questions, health histories, life events, and parents' worries and anxiety concerning the child's increased genetic risk for T1DM. Parents are also requested to complete food diaries and collect stool samples. Altogether, the TEDDY study protocol is very demanding in length and in terms of the frequency and nature of its components [1].

The collection of cord blood for screening and possible TEDDY enrollment started in September 1, 2004, and ended February 28, 2010. A total of 424,788 children were screened for increased HLA conferred genetic risk for T1DM and 21,589 were HLA eligible [11]. The enrollment rate of families with children at increased genetic risk was 38.4% from the general population (GP) and 64.8% from FDR families. The number and proportion of eligible children as well as the number of children enrolled differed both between countries and between GP and FDR families [8].

The earlier a family withdrew from the study, the fewer data points were available. For all children, demographic factors like country, gender, month of birth, FDR/GP status, and mother's age were obtained in connection with the collection of cord blood to determine eligibility for TEDDY. Data for all children enrolled in TEDDY were also available from the questionnaires that each parent completed in connection with the first TEDDY visit. These psychosocial measures contained questions on parents' views on the child's risk for developing diabetes, their worries about that possibility, and their thoughts on having the child tested for genetic T1DM risk. The mother also answered questions on lifestyle factors during pregnancy (smoking and alcohol consumption). For families withdrawing after the first study year, there were additional demographic data collected during the first year of TEDDY, including parents' education, child ethnic minority status, only child status, and household crowding. Psychosocial data were collected repeatedly starting at the 3 and 6 months' visits, at 15 months, at 27 months, and yearly. In the current study, the psychosocial data collected immediately before withdrawal were used. These data are worries (e.g.,* How often do you worry that your child will get diabetes: never/very often*), anxiety (*short version of the State-Trait Anxiety Inventory* (*SAI*)) [12], depression (*Bradley's Wellbeing Scale*) [13], study satisfaction (*three correlated questions summed into a satisfaction score: Overall, how do you feel about having your child participate in the TEDDY study? Do you think your child's participation in the TEDDY study was a good decision? Would you recommend the TEDDY study to a friend?*), and risk perception (*Compared to other children do you think your child's risk to develop diabetes is much lower/much higher?*).

### 2.2. Data Collection

When a family withdrew from TEDDY, a Change in Study Participation Form (CSP Form) was filled out by TEDDY staff and any reason the parent gave for leaving the study was recorded. More than one reason could be recorded. If a family did not give a reason for leaving the study this was also noted. The last visit when any data were collected from the family was taken as the time of withdrawal even though the CSP form could be completed at a much later date. For some families, the decision to withdraw from TEDDY was difficult and could span over a long period of time with several cancelled visits and no collection of data before a final decision to leave the study. If a family did not come to the clinic for more than a year despite scheduled visits and did not contribute any data over the course of one year, the family was considered to be a Passive Withdrawal (PW) and a CSP Form was completed. Families that became unavailable and impossible to reach were also PW and were considered TEDDY withdrawals.

### 2.3. Statistical Analysis

Differences in frequencies between categorical groups were tested by chi-square tests. For continuous variables, differences in means were tested by independent two-sample *t*-tests. Multiple linear regression was used to examine the association of demographic and psychosocial variables with specific common reasons for withdrawing. Demographic variables available on all subjects were examined first and later psychosocial factors were added. Data from the last questionnaire prior to withdrawal were used to estimate maternal anxiety, risk perception, worry, and study satisfaction. If for any reason there were missing data, information was taken from the last questionnaire available. Univariate and multiple logistic regression models were used to test for significant factors associated with type of withdrawal (PW versus AW). Analyses were performed using SAS 9.2. *p* values less than 0.05 were considered statistically significant.

## 3. Results

**Figure 1:**
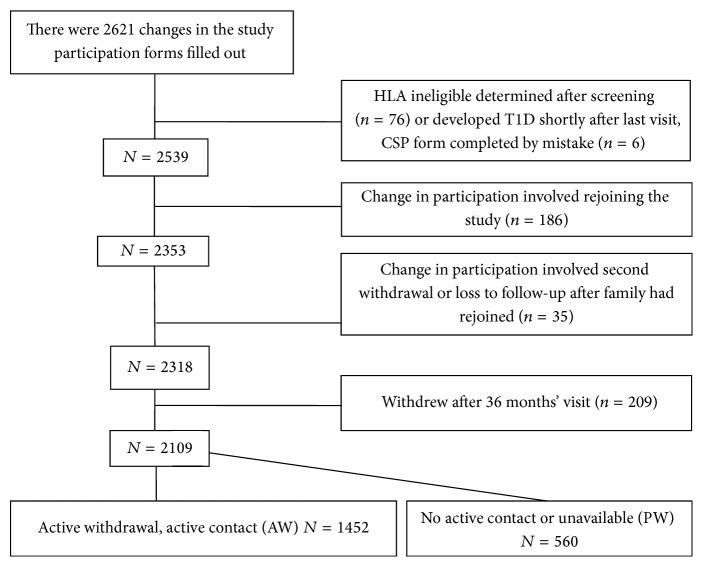
Study population.

**Figure 2:**
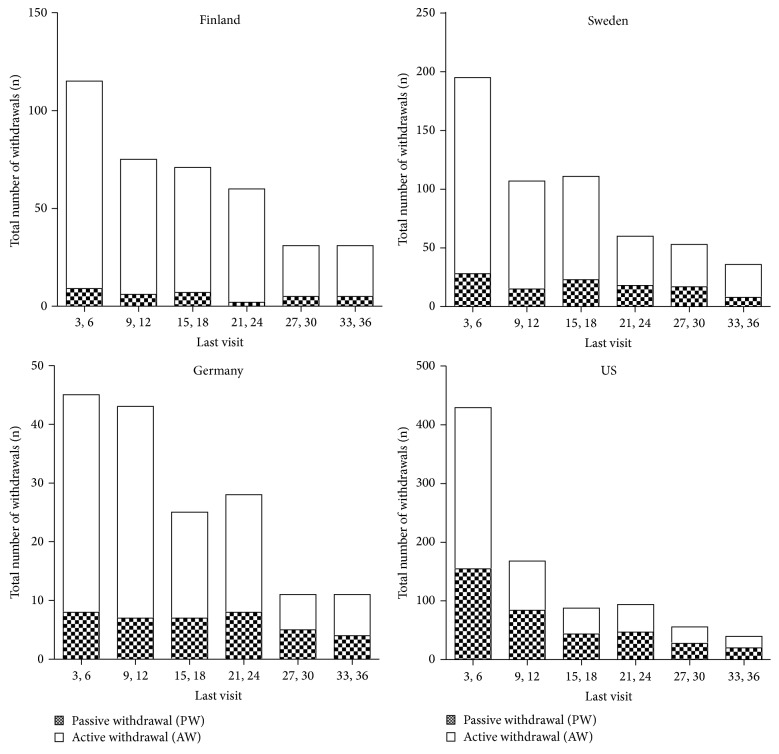
Number of withdrawals by age of last visit and by country divided into passive withdrawals (PW) (checked) and active withdrawals (AW) (white).

A total of 8677 children were recruited into the TEDDY study. From September 1, 2004, until July 31, 2012, there were 2621 CSP forms filled out. CSP forms from all children who left the study on or before the 3-year visit were selected for analysis.  Figure 1 gives an overview of how the study cohort was created. In all, 512 forms were excluded from the study for reasons outlined in the figure, resulting in 2109 CSP forms describing the first time withdrawal of the family from the study. Of these, 1549 forms came from families who told the staff they wanted to withdraw from TEDDY (AW); 90% gave at least one reason why they opted out. A total of 560 families (26.6%) did not respond to repeated scheduling attempts for more than a year or became impossible to contact and were withdrawn by the TEDDY staff without any further contact (PW). In Figure 2, the number of AW and PW for the different countries is shown by visit. Overall, study withdrawal was highest during the first year of the study and decreased thereafter and AW was far more common than PW. The United States had the highest frequency of PW (*n* = 378; 23.9%) and the relative proportion of PW increased over time. Finland had the lowest PW rate (*n* = 34; 8.9%) and it remained low across all study years. In Germany 39 children (23.9%) and in Sweden 109 children (19.0%) were classified as PW. There was a significantly increasing trend of PW proportion over the study period in Germany (*p* = 0.024) and Sweden during the three years (*p* = 0.001) even though the number of both PW and AW decreased.

Univariate and multiple regressions were used to identify factors that differentiated between AW and PW. PW were significantly more common in other countries compared to Finland, among young moms, and in older children. In the univariate models, high anxiety, maternal smoking during pregnancy, and lack of father participation in TEDDY were associated with PW. However, these factors did not remain significant in the multivariate model (Table 1).

Table 2 depicts characteristics of the AW by age of the child at the time of withdrawal. In the first half year after enrollment, 24.1% of the AW were young mothers (<25 years) which was significantly different compared to year 3 when 14.8% were young mothers. The early AW were also more anxious mothers (<0.001) and mothers more worried about their child developing diabetes (*p* = 0.003) compared to those remaining in the study. There were no significant associations between the accuracy of the mother's T1DM risk perception or study satisfaction and the child's age at withdrawal.

The frequencies of different reasons reported by the AW distributed over visits during the three study years are shown in Table 3. The different reasons are grouped into “protocol characteristics” or “family factors.” The two reasons most frequently given for leaving TEDDY were concerns about the blood draw (*n* = 359; 55% of all protocol characteristics) and being too busy/not having enough time (*n* = 587; 66.6% of all family factors). Other frequently mentioned protocol characteristics included the following: the protocol is too demanding, transportation difficulties, and the frequency of visits. Among the family factors, feeling overwhelmed/being too stressed is the second most common reason given (*n* = 206, 23.4%). Having concerns about the blood draw was more often mentioned for older children (*p* = 0.039), not wanting to be reminded of the child's risk was significantly more often reported as a reason for withdrawal during the first visits (*p* = 0.003), and being too busy/not having enough time was more often reported in the later visits (*p* = 0.037). During the study period, 11% (*n* = 97) of the families cited moving out of the area as a reason for opting out. A total of 165 families (10.7%) did not want to give a reason for leaving or only wanted to wait and see what might happen.

The result of multiple regressions examining demographic factors in all AW subjects for the two most often mentioned reasons for withdrawal (concerns about blood draw and being too busy) is presented in Table 4. German and US mothers were more likely to report the blood draw as the reason for leaving TEDDY compared to Finland and Sweden. Also, the blood draw was mentioned more often as the child got older and if the child was a girl. Being too busy was given as the reason for leaving TEDDY most often among Swedish mothers and least often among German mothers. This reason was more common in families with an older TEDDY child and if the child was a boy. Maternal smoking during pregnancy, mother working during pregnancy, maternal alcohol consumption during pregnancy, father participation in TEDDY, and whether the TEDDY child was a FDR or from the GP were not associated with either reason for leaving TEDDY.

In Table 5, two logistic regressions explore maternal psychosocial factors in relation to the two most frequently mentioned factors for withdrawing, concerns about blood draw and being too busy. The regressions are adjusted for country of residence, maternal age, age of child at study withdrawal, and gender. The results show that concerns about blood draw were associated with the mother's study satisfaction both at 6 months and at the last visit before withdrawal. At both 6 months and the last visit, mothers who reported less satisfaction with TEDDY were more likely to report concerns about the blood draw as the reason for leaving TEDDY. The relationship between study satisfaction and mothers' report of being too busy as the reason to leave TEDDY was less clear. No other psychosocial factors (maternal anxiety or mother's risk perception) showed an association with the two most common reasons for leaving the TEDDY study.

## 4. Discussion

The TEDDY study, which seeks to identify factors associated with the development of T1DM, has a demanding protocol for both the children and their parents. The study is also longitudinal with four visits to a TEDDY clinic each year until the child is four years of age and biannually thereafter until the child is fifteen years of age. After 8 years, 72.2% of the recruited children are still participating in the study. The majority of families who left gave a reason for leaving. PW was more common among the US participants. Being a large country with a diverse population, it is more difficult to track people compared to the European countries. We previously reported that U.S. families often failed to respond to phone messages or letters inviting them to join TEDDY [8], constituting passive refusal, which is similar to PW.

Finland had the lowest number of PW and similar numbers over the years, while the proportion of PW in Germany and Sweden tended to increase during the three study years even though the total number of withdrawals decreased significantly. Some study sites may keep a TEDDY participant as “active” in TEDDY despite multiple missed visits. After getting to know TEDDY staff over many months some families may have difficulty directly telling staff that they are leaving the study and may instead just “no-show.” Differences between study centers in how families are managed might develop over time and this is a weakness of the current results reported. However, it is difficult for a large study like TEDDY to systematically define how staff uniformly manage study families over many years.

Sociodemographic factors also related to study withdrawal. PW mothers were younger and more likely to smoke during pregnancy than those retained in the study. In the univariate analysis, lack of father participation in TEDDY was associated with PW but this effect was not statistically significant in the multivariate model. In previous work, we found that lack of father participation was an important predictor of study withdrawal in the first year of TEDDY [9]. Father involvement in a study may be a more important determinant of whether a family stays in a study or withdraws; it may not predict whether the withdrawal is active or passive.

Logistical aspects of the study were found to be common reasons for withdrawal. Even though TEDDY staff is very skilled, drawing blood from a small child can be very challenging and cause unpleasant experiences both for the child and the parent. Other studies have reported that blood draws can be an obstacle for study participation and a reason for opting out [7]. Reporting the blood draw as a reason for leaving TEDDY was more common later in the study than in the early phase. All blood draws are done after application of dermal anesthetics so a baby might react less than a slightly older child who might have learned to fear the blood draw. This observation has been verified by Swedish TEDDY nurses who conducted a parent survey of the child's reaction to the blood draw. In fact, parents tended to rate stronger reactions in older children (personal communication).

Being too busy and not having time to do the TEDDY tasks was the most frequently mentioned reason for leaving the study. Being stressed and feeling overwhelmed was another important reason for not participating anymore. Being busy was significantly more often mentioned as a reason for withdrawal at the later study visits. It may be that when a baby is born, the mother is often home caring for the baby and may not experience the TEDDY tasks as burdensome, compared to later when she may return to work. Also, some TEDDY tasks are easier to complete when the child is a baby like collecting stool samples or doing a 3-day food diary. Also, being in the beginning of a study may give participants a feeling of curiosity and enthusiasm, something that may disappear as the study seems less novel to families.

Psychological reasons also played a role in withdrawal for some families, particularly early in the study. Mothers in families who withdrew early, after the first or second TEDDY visit, appeared to be more anxious and worried about their child getting diabetes compared to mothers in families leaving the study at 9 months or later. This is underscored by the observation that mothers who reported that they did not want to be reminded of the child's risk of T1DM as a reason for withdrawal often left TEDDY after the first two visits.

The two most important factors mentioned as reasons for withdrawal (blood draw and being too busy) were each analyzed in separate regression models, first in relation to demographic factors and in a second model in relation to maternal psychological factors and study satisfaction. Reporting the blood draw as a reason for withdrawal was more common if the child was a girl while stating that the family was too busy to participate was more common if the child was a boy. A study on infant pain response following immunization injection demonstrated that parental behavior has a key role in influencing how infants respond to painful procedures with differences between female and male infants [14]. We can only speculate that parents may be more sensitive to the possible discomfort of the blood draw in girls than in boys; boys are often expected to be braver than girls. Another study found that girls' pain threshold is lower than that of boys, at least for slightly older children compared to the TEDDY children in our study [15]. This could indicate that the reaction of girls to the blood draw might be stronger than that of the boys and therefore the parents might be more prone to opt out when the child is a girl even when the child is younger.

Why being too busy was more often mentioned when the child was a boy is harder to explain. Sometimes boys are more physically active and this may create more problems for parents in collecting TEDDY samples and data in preparation for the visit. It may also mean that boys are more likely to openly protest going to the TEDDY clinic. All this taken together might give parents a feeling of not having time and being too busy. In the current study, parents who gave the blood draw as a reason for leaving often expressed lower satisfaction with the study both at 6 months, when this was first assessed in TEDDY, and in the survey completed before opting out. It is likely that difficulties with the blood draw were seen early in the study, sometimes continued, and resulted in lower satisfaction with the TEDDY experience and ultimately withdrawal from the study.

In a prior study exploring reasons of why parents stay in TEDDY, having someone watching the child for development of T1DM was the most often mentioned reason. Among the minority of parents who had considered leaving the study, the blood draw, being too busy/not having enough time, a demanding protocol, and food diaries were the most frequently reported reasons for considering leaving [16]. These results are in line with what was found in the present study.

In this study, reasons for withdrawal were obtained by the TEDDY staff via interview when the parents decided to leave TEDDY, while our prior published work collected this information by questionnaire [16]. Therefore, the method of obtaining this data does not seem to be important as the results were similar. What is lacking is a more in-depth explanation of why parents are too busy and do not have time to remain in TEDDY. Retention and compliance in a longitudinal study like TEDDY are critical for the success of the study so detailed information about why families leave is important for developing strategies for improving study retention.

## 5. Conclusion

Results from this study suggest some significant factors that should be taken into account to counteract opting out in longitudinal studies focusing on a population at genetic risk for T1DM like TEDDY. Psychological factors clearly play a role in early withdrawal and thus early in the study it is important to record and pay attention to parents' anxieties and worries and to implement procedures that may reduce or address these challenges. Also, young mothers, particularly if the father is not fully present, are at early risk for leaving the study and may need extra attention. Procedures that can be experienced as painful and frightening, like a venous blood draw, need to be used with great caution and all ways to facilitate obtaining the specimen need to be considered. It is important for researchers to carefully think through all components in the study that might increase the demands on the participants and it is important to avoid overburdening families, which may increase the risk of withdrawal. Regular investigations of the subjects' satisfaction with the study can give important information on how the study subjects are experiencing their participation and can predict withdrawal.

## Figures and Tables

**Table 1:** Factors associated with passive withdrawal (PW) versus active withdrawal (AW).

Factors^a^	Numbermean (SD)	Univariate				Multivariate		
		% of PW	OR	95% CI	*p* value	OR	95% CI	*p* value
Country of residence								
Finland	383	8.9	1.00	Ref.		1.00	Ref.	
Sweden	562	19.4	2.47	1.64–3.72		2.86	1.85–4.44	
Germany	163	23.9	3.22	1.95–5.34		3.73	2.16–6.43	
US	1001	37.8	4.28	3.26–9.06	<0.001	6.57	4.35–9.93	<0.0011
Maternal age at child's birth (years)								
Years	28.9 (5.7)		0.94	0.93–0.96	<0.001	0.95	0.93–0.97	<0.001
<29 years	1016	32.0						
≥29 years	1093	21.5						
Child's age of withdrawal (years)								
Years	1.16 (0.80)		1.12	0.99–1.25	0.08	1.33	1.16–1.52	<0.001
<1.16 years	1231	25.8						
≥1.16 years	878	27.6						
Gender								
Female	1094	25.9	1.00	Ref.		1.00	Ref.	
Male	1015	27.3	1.08	0.88–1.31	0.46	1.10	0.88–1.36	0.40
FDR^a^								
No	1957	26.8	1.00	Ref.		1.00	Ref.	
Yes	152	23.7	0.85	0.57–1.25	0.41	0.95	0.62–1.47	0.81
Smoking during pregnancy								
No	1556	25.2	1.00	Ref.		1.00	Ref.	
Yes	415	30.4	1.30	1.02–1.64	0.03	1.31	1.00–1.72	0.05
Alcohol 3rd trimester								
No	1713	26.1	1.00	Ref.		1.00	Ref.	
Yes	289	27.3	1.07	0.81–1.41	0.66	1.16	0.85–1.60	0.35
Worked during pregnancy								
No or reduced hours	1184	26.6	1.00	Ref.		1.00	Ref.	
Yes or increased hours	819	25.8	0.96	0.78–1.17	0.67	0.92	0.74–1.15	0.45
High anxiety (SAI > 48)								
Score	40.9 (10.7)		1.02	1.01–1.03	<0.001	1.00	0.99–1.01	0.80
<40.9 score	1059	22.6						
≥40.9 score	921	29.4						
Risk perception								
Underestimate	901	27.3	1.00	Ref.		1.00	Ref.	
Accurate	1097	25.2	0.90	0.73–1.09	0.28	0.99	0.80–1.23	0.93
Father not active (3 mo)^b^								
No	1807	25.0	1.00	Ref.		1.00	Ref.	
Yes	302	36.1	1.70	1.31–2.20	<0.001	1.27	0.90–1.78	0.17

^a^FDR = first degree relative has type 1 diabetes. ^b^Father did not answer 3-month questionnaires.

**Table 2:** Active withdrawals by child's age of withdrawal and demographic factors, maternal psychosocial factors, and maternal study satisfaction.

Factors	All 3–36 m (*N*)	Year 1 3–6 m *N* (row %) or mean (SD)	Year 1 9–12 m *N* (row %) or mean (SD)	Year 2 15–24 m *N* (row %) or mean (SD)	Year 3 27–36 m *N* (row %) or mean (SD)	*p* value
Number of active withdrawals	1549	584 (37.7)	324 (20.9)	431 (27.8)	210 (13.6)	
Country of residence						
Finland	349	106 (30.4)	69 (19.8)	122 (35.0)	52 (14.9)	
Sweden	453	167 (36.9)	92 (20.3)	130 (28.7)	64 (14.1)	
Germany	124	37 (29.8)	36 (29.0)	38 (30.6)	13 (10.5)	
US	623	274 (44.0)	127 (20.4)	141 (22.6)	81 (13.0)	<0.001
Gender						
Female	811	323 (39.8)	159 (19.6)	232 (28.6)	97 (12.0)	
Male	738	261 (35.4)	165 (22.4)	199 (27.0)	113 (15.3)	0.07
First degree relative with T1D						
No	1433	534 (37.3)	302 (21.1)	406 (28.3)	91 (13.3)	
Yes	116	50 (43.1)	22 (19.0)	25 (21.6)	19 (16.4)	0.30
Maternal age at child's birth						
years	29.4 (5.6)	28.9 (5.8)	29.0 (5.2)	29.6 (5.5)	30.9 (5.5)	<0.001
Highly anxious at last visit (SAI > 48)^a^						
No	1241	413 (33.3)	259 (20.9)	375 (30.5)	191 (15.4)	
Yes	229	116 (50.7)	55 (24.0)	45 (19.7)	13 (5.7)	<0.001
Worry about diabetes at last visit^a,b^						
Never or rarely	706	104 (14.7)	166 (23.5)	290 (41.4)	144 (20.4)	
Sometimes or very often	344	56 (16.3)	114 (33.1)	113 (33.4)	59 (17.2)	0.004
Risk perception at last visit^a^						
Underestimate	692	247 (35.7)	147 (21.2)	207 (30.1)	90 (13.0)	
Accurate	783	283 (36.1)	170 (21.7)	213 (27.5)	115 (14.7)	0.63
Study satisfaction at last visit^a,b^						
Very satisfied	276	38 (13.8)	77 (27.9)	108 (39.1)	53 (19.2)	
Satisfied	289	44 (15.2)	87 (30.1)	99 (34.3)	59 (20.4)	
Somewhat satisfied	274	39 (14.2)	71 (25.9)	115 (42.0)	49 (17.9)	
Neutral or dissatisfied	208	38 (18.3)	44 (21.2)	84 (40.4)	42 (20.2)	0.47

^a^If no measure last visit, the second to last visit is taken if available.

^b^Study satisfaction and worry about diabetes are not asked in the first questionnaires so there are fewer available answers at 3 and 6 months.

**Table 3:** Frequency of common reasons for withdrawing from the TEDDY study during the first three study years. Percentages for reasons mentioned more frequently are shown.

Withdrawals	Last visit before withdrawal (month)					
	Year 1	Year 1	Year 2	Year 3	All	
	3–6 m	9–12 m	15–24 m	27–36 m	3–36 m	
	*N* (row %)	*N* (row %)	*N* (row %)	*N* (row %)	*N*	%
Active withdrawals (AW)	**584 (37.7)**	**324 (20.9)**	**431 (27.8)**	**210 (13.6)**	**1549**	**73.4**
Reasons for withdrawal						
**Protocol characteristics**	**244 (37.4)**	**128 (19.6)**	**195 (29.9)**	**86 (13.2)**	**653**	**100**
Concerns about blood draw	124 (34.5)	63 (17.5)	112 (31.2)	60 (16.7)	359	55.0^a^
Protocol too demanding	68 (40.0)	41 (24.1)	43 (25.3)	18 (10.6)	170	26.0^a^
Transportation difficulties	44 (41.1)	21 (19.6)	35 (32.7)	7 (6.5)	107	16.4
Concerns about frequency of visits	40 (39.2)	20 (19.6)	33 (32.4)	9 (8.8)	102	15.6
Concerns about stool samples	14	9	13	7	43	6.6
Concerns about questionnaires	15	8	10	4	37	5.7
Food diaries too troublesome	12	1	2	1	34	5.2
Do not want to be reminded of risk	19	6	5	1	31	4.7
Duration of study is too long	1	3	5	1	10	1.5
No treatment to prevent offered	3	1	2	1	7	1.1
Worried about privacy	1	1	0	0	2	—
Worried about loss of insurance	3	0	0	0	3	—
Other protocol characteristics	2	3	5	2	12	1.8
**Family factors**	**279 (35.6)**	**162 (20.7)**	**231 (29.5)**	**112 (14.3)**	**784**	**100**
Too busy/not enough time	187 (31.9)	134 (22.8)	185 (31.5)	81 (13.8)	587	66.6^a^
Feeling overwhelmed/stressed	82 (39.8)	40 (19.4)	58 (28.2)	26 (12.6)	206	23.4
Moving out of the study area	33 (30.4)	23 (23.7)	22 (22.7)	19 (19.6)	97	11.0
Child medical/behavioral problems	29 (40.3)	16 (22.2)	16 (22.7)	11 (19.6)	72	8.2
Family member emotional problems	15	9	18	10	52	5.9
Does not want to be in research	9	3	8	5	25	2.8
Family member does not agree to participate	8	0	5	4	17	1.9
Family member in another study	1	0	2	0	3	—
Subject already in another study	0	1	0	0	1	—
Fam. health care provider not recommended	1	0	0	0	1	—
Language barrier	1	0	0	0	1	—
Other family factors	4	2	4	3	13	1.5
**Active contact made but no reason given or ** **wants to wait and see **	**70 (42.4)**	**28 (17.0)**	**42 (25.4)**	**25 (15.2)**	**165**	**10.7**

^a^Significant difference between age groups: concerns about blood draw (*p* = 0.039), do not want to be reminded of risk (*p* = 0.003), and too busy/not enough time (*p* = 0.037).

**Table 4:** Multiple logistic regression examining demographic factors in relation to (a) concerns about blood or (b) being too busy as a reason for withdrawing among those who actively withdrew.

Factors at enrollment	Demographic measures in relation to concerns about blood draw and being too busy (*n* = 1549)					
	(a) Concerns about blood draw			(b) Being too busy		
	OR	95% CI	*p* value	OR	95% CI	*p* value
Country of residence						
Finland	1.00	Ref.		1.00	Ref.	
Sweden	1.33	0.91–1.93		1.47	1.10–1.96	
US	1.72	1.21–2.43		0.83	0.63–1.09	
Germany	7.80	4.89–12.4	<0.001	0.48	0.30–0.77	<0.001
Maternal age at child's birth (yrs)						
Years	1.05	1.02–1.07	<0.001	1.00	0.98–1.01	0.62
Child's age of withdrawal (months)						
Months	1.20	1.03–1.40	0.02	1.15	1.01–1.31	0.04
Gender						
Male	1.00	Ref.		1.00	Ref.	
Female	1.44	1.12–1.85	0.004	0.78	0.63–0.96	0.02

Note: FDR/GP status, smoking during pregnancy, working during pregnancy, alcohol consumption during pregnancy, and dad's participation in TEDDY were not associated with either concerns about blood draw or being too busy.

**Table 5:** Logistic regression examining maternal psychosocial factors in relation to (a) concerns about blood draw or (b) being too busy as a reason for withdrawing after adjusting for country of residence, maternal age, child's age at withdrawal, and gender (see Table 4).

Psychosocial factors	Psychosocial measures in relation to concerns about blood and being too busy after adjusting for demographic factors (*n* ~ 1031)					
	(a) Concerns about blood draw			(b) Being too busy		
	OR	95% CI	*p* value	OR	95% CI	*p* value
Maternal high anxiety (SAI > 48)						
Score	1.00	0.98–1.01	0.42	0.99	0.98–1.00	0.09
Mother's risk perception						
Underestimate	1.00	Ref.		1.00	Ref.	
Accurate	1.01	0.74–1.38	0.95	0.91	0.70–1.18	0.49
Study satisfaction (6 mo)						
Very satisfied	1.00	Ref.		1.00	Ref.	
Satisfied	1.38	0.89–2.15		1.24	0.88–1.74	
Somewhat satisfied	1.80	1.16–2.78		1.80	1.27–2.59	
Neutral or dissatisfied	2.73	1.68–4.42	<0.001	1.11	0.73–1.69	0.007
Study satisfaction (last visit)						
Very satisfied	1.00	Ref.		1.00	Ref.	
Satisfied	1.57	0.99–2.49		1.19	0.84–1.69	
Somewhat satisfied	2.54	1.61–4.03		1.43	1.00–2.05	
Neutral or dissatisfied	2.64	1.62–4.31	<0.001	1.33	0.89–1.97	0.252

## Appendix

### The TEDDY Study Group

*Colorado Clinical Center*. Marian Rewers, M.D., Ph.D., PI (Ancillary Studies, Human Subjects/Publicity/Publications, Immune Markers, Infectious Agents, Quality Assurance, Steering), Kimberly Bautista (Study Coordinators), Judith Baxter (Psychosocial, Quality Assurance, Study Coordinators, Quality Assurance Subcommittee on Data Quality), Ruth Bedoy (Diet), Daniel Felipe-Morales, Brigitte Frohnert, M.D., and Patricia Gesualdo (Diet, Infectious Agents, Study Coordinators, Clinical Implementation, Quality Assurance Subcommittee on Data Quality), Michelle Hoffman (Study Coordinators, Celiac Disease, Clinical Implementation), Rachel Karban (Study Coordinators), Edwin Liu, M.D. (Celiac Disease), Jill Norris, Ph.D. (Diet, Genetics, Study Coordinators), Adela Samper-Imaz and Andrea Steck, M.D. (Genetics, Clinical Implementation), Kathleen Waugh (Infectious Agents, Laboratory Implementation, Study Coordinators, Quality Assurance Subcommittee on Data Quality), and Hali Wright (Study Coordinators). University of Colorado, Anschutz Medical Campus, Barbara Davis Center for Childhood Diabetes.

*Georgia/Florida Clinical Center*. Jin-Xiong She, Ph.D., PI (Ancillary Studies, Genetics, Human Subjects/Publicity/Publications, Steering, Jinfiniti Biosciences LLC, Augusta, GA), Desmond Schatz, M.D. (University of Florida, Human Subjects/Publicity/Publications, Immune Markers, Laboratory Implementation, Maternal Studies), Diane Hopkins (Study Coordinators), Leigh Steed (Study Coordinators, Celiac Disease, Clinical Implementation, Quality Assurance Subcommittee on Data Quality), Jamie Thomas (University of Florida, Infectious Agents, Study Coordinators), Katherine Silvis (Diet), Michael Haller, M.D. (University of Florida, Clinical Implementation), Meena Shankar (University of Florida, Diet), Eleni Sheehan (University of Florida), Melissa Gardiner, Richard McIndoe, Ph.D., Haitao Liu, M.D. (Jinfiniti Biosciences LLC, Augusta, GA), John Nechtman (Jinfiniti Biosciences LLC, Augusta, GA), and Ashok Sharma, Joshua Williams, Gabriela Foghis, and Stephen W. Anderson, M.D. (Pediatric Endocrine Associates, Atlanta, GA). Medical College of Georgia, Georgia Regents University.

*Germany Clinical Center*. Anette G. Ziegler, M.D., PI (Ancillary Studies, Genetics, Human Subjects/Publicity/Publications, Steering), Andreas Beyerlein, Ph.D. (Diet), Ezio Bonifacio, Ph.D. (Center for Regenerative Therapies, TU Dresden, Immune Markers), Michael Hummel, M.D. (Celiac Disease), Sandra Hummel, Ph.D. (Diet), Kristina Foterek (Research Institute for Child Nutrition, Dortmund, Diet), Mathilde Kersting, Ph.D. (Research Institute for Child Nutrition, Dortmund, Diet), Annette Knopff (Laboratory Implementation), Sibylle Koletzko, M.D. (Dr. von Hauner Children's Hospital, Department of Gastroenterology, Ludwig Maximillians University Munich, Celiac Disease), Claudia Peplow (Study Coordinators), Roswith Roth, Ph.D. (Psychosocial), Joanna Stock (Psychosocial, Study Coordinators), Elisabeth Strauss (Study Coordinators), Katharina Warncke, M.D. (Clinical Implementation), and Christiane Winkler, Ph.D. (Diet, Study Coordinators, Quality Assurance Subcommittee on Data Quality). Forschergruppe Diabetes e.V. and Institute of Diabetes Research, Helmholtz Zentrum München, and Klinikum rechts der Isar, Technische Universität München.

*Finland Clinical Center*. Jorma Toppari, M.D., Ph.D., PI (University of Turku, Turku University Hospital, Hospital District of Southwest Finland, Ancillary Studies, Human Subjects/Publicity/Publications, Steering, Clinical Implementation), Olli G. Simell, M.D., Ph.D., PI (University of Turku, Turku University Hospital, Hospital District of Southwest Finland, Ancillary Studies, Human Subjects/Publicity/Publications, Steering, Celiac Disease), Annika Adamsson, Ph.D. (Turku University Hospital, Hospital District of Southwest Finland, Study Coordinators), Heikki Hyöty, M.D., Ph.D. (University of Tampere, Tampere University Hospital, Infectious Agents), Jorma Ilonen, M.D., Ph.D. (University of Turku, University of Kuopio, Genetics), Miia Kähönen (University of Oulu, Oulu University Hospital), Mikael Knip, M.D., Ph.D. (University of Tampere, Tampere University Hospital, Immune Markers), Annika Koivu (University of Turku, Turku University Hospital, Hospital District of Southwest Finland), Mirva Koreasalo (University of Tampere, Tampere University Hospital, National Institute for Health and Welfare, Finland, Diet), Kalle Kurppa, M.D., Ph.D. (University of Tampere, Tampere University Hospital, Celiac Disease), Maria Lönnrot, M.D., Ph.D. (University of Tampere, Tampere University Hospital, Infectious Agents), Elina Mäntymäki (University of Turku, Turku University Hospital, Hospital District of Southwest Finland), Katja Multasuo (University of Oulu, Oulu University Hospital), Juha Mykkänen, Ph.D. (Turku University Hospital, Hospital District of Southwest Finland, University of Turku, Genetics), Tiina Niininen (Tampere University Hospital, University of Tampere, Study Coordinators), Mia Nyblom (University of Tampere, Tampere University Hospital), Petra Rajala (Turku University Hospital, Hospital District of Southwest Finland), Jenna Rautanen (Tampere University Hospital, National Institute for Health and Welfare, Finland), Anne Riikonen (University of Tampere, Tampere University Hospital), Minna Romo (University of Turku, Turku University Hospital, Hospital District of Southwest Finland), Satu Simell, M.D., Ph.D. (Turku University Hospital, Hospital District of Southwest Finland, Tampere University Hospital, Celiac Disease), Tuula Simell, Ph.D., Ville Simell (Turku University Hospital, Hospital District of Southwest Finland, University of Turku, Celiac Disease), Maija Sjöberg (University of Turku, Turku University Hospital, Hospital District of Southwest Finland, Study Coordinators, Clinical Implementation), Aino Stenius (University of Oulu, Oulu University Hospital, Study Coordinators), Eeva Varjonen (University of Turku, Turku University Hospital, Hospital District of Southwest Finland, Study Coordinators), Riitta Veijola, M.D., Ph.D. (University of Oulu, Oulu University Hospital, Clinical Implementation), Suvi M. Virtanen, M.D., Ph.D. (University of Tampere, Tampere University Hospital, National Institute for Health and Welfare, Finland, Diet), and Mari Åkerlund (University of Tampere, Tampere University Hospital, National Institute for Health and Welfare, Finland).

*Sweden Clinical Center*. Åke Lernmark, Ph.D., PI (Ancillary Studies, Genetics, Human Subjects/Publicity/Publications, Immune Markers, Infectious Agents, Maternal Studies, Quality Assurance, Steering, Quality Assurance Subcommittee on Data Quality), Daniel Agardh, M.D., Ph.D. (Celiac Disease), Carin Andrén Aronsson (Diet, Celiac Disease), Maria Ask, Jenny Bremer, Ulla-Marie Carlsson, and Corrado Cilio, Ph.D., M.D. (Immune Markers), Camilla Ekstrand and Emelie Ericson-Hallström (Diet), Lina Fransson, Thomas Gard, Joanna Gerardsson, Rasmus Håkansson, Monica Hansen, and Gertie Hansson (Study Coordinators), Susanne Hyberg, Fredrik Johansen, Berglind Jonasdottir, M.D., Linda Jonsson, and Helena Elding Larsson, M.D., Ph.D. (Infectious Agents, Clinical Implementation), Barbro Lernmark, Ph.D., Maria Månsson-Martinez, Maria Markan, Theodosia Massadakis, and Jessica Melin (Study Coordinators), Zeliha Mestan, Kobra Rahmati, Anita Ramelius, Falastin Salami, Monica Sedig Järvirova, Sara Sibthorpe, Birgitta Sjöberg, and Ulrica Swartling, Ph.D. (Psychosocial, Study Coordinators), Erika Trulsson and Carina Törn, Ph.D. (Genetics, Quality Assurance Subcommittee on Data Quality), Anne Wallin and Åsa Wimar (Study Coordinators, Clinical Implementation), and Sofie Åberg. Lund University.

*Washington Clinical Center*. William A. Hagopian, M.D., Ph.D., PI (Ancillary Studies, Genetics, Human Subjects/Publicity/Publications, Immune Markers, Infectious Agents, Laboratory Implementation, Steering, Celiac Disease, Clinical Implementation), Xiang Yan, M.D., and Michael Killian (Infectious Agents, Laboratory Implementation, Study Coordinators, Celiac Disease), Claire Cowen Crouch (Study Coordinators, Clinical Implementation, Quality Assurance Subcommittee on Data Quality), Jennifer Skidmore (Diet), Stephen Ayres, Kayleen Dunson, Diana Heaney, Rachel Hervey, Corbin Johnson, Rachel Lyons, Arlene Meyer, Denise Mulenga, Emma Schulte, Elizabeth Scott, Joshua Stabbert, and John Willis. Pacific Northwest Diabetes Research Institute.

*Pennsylvania Satellite Center*. Dorothy Becker, M.D., Margaret Franciscus, and MaryEllen Dalmagro-Elias Smith (Diet), Ashi Daftary, M.D., Mary Beth Klein, and Chrystal Yates. Children's Hospital of Pittsburgh of UPMC.

*Data Coordinating Center*. Jeffrey P. Krischer, Ph.D., PI (Ancillary Studies, Human Subjects/Publicity/Publications, Immune Markers, Quality Assurance, Steering), Michael Abbondondolo, Sarah Austin-Gonzalez, and Rasheedah Brown (Study Coordinators, Quality Assurance Subcommittee on Data Quality), Brant Burkhardt, Ph.D. (Immune Markers, Infectious Agents), Martha Butterworth (Diet), David Cuthbertson, Christopher Eberhard, Steven Fiske (Psychosocial), Dena Garcia, Veena Gowda, David Hadley, Ph.D. (Genetics, Celiac Disease), Hye-Seung Lee, Ph.D. (Ancillary Studies, Diet, Celiac Disease, Quality Assurance Subcommittee on Data Quality), Shu Liu and Xiang Liu, Ph.D. (Diet, Psychosocial, Study Coordinators), Kristian Lynch, Ph.D. (Immune Markers, Infectious Agents, Psychosocial, Quality Assurance Subcommittee on Data Quality), Jamie Malloy and Cristina McCarthy (Study Coordinators, Quality Assurance Subcommittee on Data Quality), Wendy McLeod (Diet, Immune Markers, Infectious Agents, Celiac Disease, Quality Assurance Subcommittee on Data Quality), Chris Shaffer and Laura Smith, Ph.D. (Psychosocial, Study Coordinators), Susan Smith (Study Coordinators, Quality Assurance Subcommittee on Data Quality), Roy Tamura, Ph.D. (Ancillary Studies, Diet, Celiac Disease), Ulla Uusitalo, Ph.D. (Diet, Quality Assurance Subcommittee on Data Quality), Kendra Vehik, Ph.D. (Human Subjects/Publicity/Publications, Immune Markers, Infectious Agents, Clinical Implementation, Quality Assurance Subcommittee on Data Quality), and Ponni Vijayakandipan, Keith Wood, and Jimin Yang, Ph.D., R.D. (Diet, Quality Assurance Subcommittee on Data Quality). University of South Florida.

*Project Scientist*. Beena Akolkar, Ph.D. (Ancillary Studies, Genetics, Human Subjects/Publicity/Publications, Immune Markers, Infectious Agents, Laboratory Implementation, Quality Assurance, Steering). National Institutes of Diabetes and Digestive and Kidney Diseases.

*Other Contributors*. Kasia Bourcier, Ph.D. (Immune Markers), National Institutes of Allergy and Infectious Diseases. Thomas Briese, Ph.D. (Infectious Agents, Quality Assurance Subcommittee on Data Quality), Columbia University. Suzanne Bennett Johnson, Ph.D. (Psychosocial, Study Coordinators), Florida State University. Steve Oberste, Ph.D. (Infectious Agents), Centers for Disease Control and Prevention. Eric Triplett, Ph.D. (Infectious Agents), University of Florida.

*Autoantibody Reference Laboratories*. Liping Yu, M.D. (Barbara Davis Center for Childhood Diabetes, University of Colorado Denver, Immune Markers), Dongmei Miao, M.D. (Barbara Davis Center for Childhood Diabetes, University of Colorado Denver), Polly Bingley, M.D., FRCP (School of Clinical Sciences, University of Bristol, UK, Immune Markers), Alistair Williams (School of Clinical Sciences, University of Bristol, UK), Kyla Chandler (School of Clinical Sciences, University of Bristol, UK), Saba Rokni (School of Clinical Sciences, University of Bristol, UK), Joanna Boldison (School of Clinical Sciences, University of Bristol, UK), Jacob Butterly (School of Clinical Sciences, University of Bristol, UK), Gabriella Carreno (School of Clinical Sciences, University of Bristol, UK), Claire Caygill (School of Clinical Sciences, University of Bristol, UK), Ivey Geoghan (School of Clinical Sciences, University of Bristol, UK), Anna Long (School of Clinical Sciences, University of Bristol, UK), Molly Payne (School of Clinical Sciences, University of Bristol, UK), James Pearson (School of Clinical Sciences, University of Bristol, UK), Sophie Ridewood (School of Clinical Sciences, University of Bristol, UK), and Rebecca Wyatt (School of Clinical Sciences, University of Bristol, UK).

*Cortisol Laboratory*. Elisabeth Aardal Eriksson, M.D., Ph.D., Ing-Marie Lundgren, Ewa Lönn Karlsson, Dzeneta Nezirevic Dernroth, Ph.D. Department of Clinical Chemistry, Linköping University Hospital, Linköping, Sweden.

*Dietary Biomarkers Laboratory*. Iris Erlund, Ph.D. (Diet), Irma Salminen, Jouko Sundvall, Jaana Leiviskä, and Mari Lehtonen, Ph.D. National Institute for Health and Welfare, Helsinki, Finland.

*HbA1c Laboratory*. Randie R. Little, Ph.D., Alethea L. Tennill. Diabetes Diagnostic Laboratory, Department of Pathology, University of Missouri School of Medicine.

*HLA Reference Laboratory*. Henry Erlich, Ph.D. (Genetics), Steven J. Mack, Ph.D., Anna Lisa Fear. Center for Genetics, Children's Hospital Oakland Research Institute.

*Metabolomics Laboratory*. Oliver Fiehn, Ph.D., Bill Wikoff, Ph.D., Brian Defelice, Dmitry Grapov, Ph.D., Tobias Kind, Ph.D., Mine Palazoglu, Luis Valdiviez, Benjamin Wancewicz, Gert Wohlgemuth, and Joyce Wong. UC Davis Metabolomics Center.

*Microbiome and Viral Metagenomics Laboratory*. Joseph F. Petrosino, Ph.D. (Infectious Agents). Alkek Center for Metagenomics and Microbiome Research, Department of Molecular Virology and Microbiology, Baylor College of Medicine.

*OGTT Laboratory*. Santica M. Marcovina, Ph.D., Sc.D., Vinod P. Gaur, Ph.D., Northwest Lipid Metabolism and Diabetes Research Laboratories, University of Washington.

*Proteomics Laboratory*. Richard D. Smith, Ph.D., Thomas O. Metz, Ph.D., Charles Ansong, Ph.D., Bobbie-Jo Webb-Robertson, Ph.D., and Hugh D. Mitchell, Ph.D. Pacific Northwest National Laboratory.

*Repository*. Heather Higgins and Sandra Ke. NIDDK Biosample Repository at Fisher BioServices.

*RNA Laboratory and Gene Expression Laboratory*. Jin-Xiong She, Ph.D., PI (Ancillary Studies, Genetics, Human Subjects/Publicity/Publications, Steering), Richard McIndoe, Ph.D., Haitao Liu, M.D., John Nechtman, Yansheng Zhao, Na Jiang, M.D. Jinfiniti Biosciences, LLC.

*SNP Laboratory*. Stephen S. Rich, Ph.D. (Genetics), Wei-Min Chen, Ph.D. (Genetics), Suna Onengut-Gumuscu, Ph.D. (Genetics), Emily Farber, Rebecca Roche Pickin, Ph.D., Jordan Davis, Dan Gallo, Jessica Bonnie, and Paul Campolieto. Center for Public Health Genomics, University of Virginia.
